# Hypercalcemia in pregnancy secondary to pathogenic variants in *CYP24A1*

**DOI:** 10.1177/1753495X251391513

**Published:** 2025-12-08

**Authors:** Jocelyn Yee Ping Wong, Ellen Anne Miles

**Affiliations:** 1 Department of Internal Medicine, 12358 University of British Columbia, Vancouver, BC, Canada; 2 Department of Obstetric Internal Medicine, 8163 BC Women's Hospital, Vancouver, BC, Canada; 3 Department of General Internal Medicine, Royal Columbian Hospital, Vancouver, BC, Canada

**Keywords:** Hypercalcemia, hypercalcemia in pregnancy, *CYP24A1* deficiency, 24-hydroxylase deficiency, pregnancy

## Abstract

Hypercalcemia is rare in pregnancy and carries a significant risk for both mother and fetus. A rare cause of hypercalcemia is loss-of-function mutations in *CYP24A1*, which encodes 24-hydroxylase, responsible for the inactivation of active vitamin D metabolites. Pregnancy-associated upregulation of 1α-hydroxylase, increased parathyroid hormone (PTH)-related peptide, and supplementation with vitamin D can unmask *CYP24A1* deficiency. This condition should be suspected in women presenting with unexplained hypercalcemia, suppressed PTH, and a negative secondary workup for malignancy, granulomatous disease, and contributory medications. Management includes intravenous fluids, calcitonin, and avoidance of vitamin D and calcium supplements. Here, we report a case of persistent hypercalcemia despite aggressive hydration and calcitonin therapy. Postpartum genetic testing confirmed a homozygous *CYP24A1* mutation, with markedly elevated 1,25(OH)_2_D levels. *CYP24A1* mutations are a rare but important cause of gestational hypercalcemia. Early recognition and multidisciplinary management can improve maternal and neonatal outcomes.

## Introduction

Calcium is an essential nutrient in pregnancy, supporting fetal bone mineralization and maternal bone health.^1^ Intestinal absorption increases twofold in pregnancy, and this process is facilitated by maternal calciotropic hormones, parathyroid hormone (PTH), PTH-related peptide (PTHrP) synthesized in the breast and placenta**,** and renal upregulation of 1,25(OH)_2_D by 1α-hydroxylase.^1,2^

Hypercalcemia in pregnancy is rare and pathologic. It is associated with adverse maternal outcomes, including nephrolithiasis, pancreatitis, gestational hypertension, and pre-eclampsia, as well as adverse fetal outcomes, including fetal intrauterine growth restriction, preterm labor, stillbirth, and fetal death.^1,3,4^ Maternal hypercalcemia may cause fetal parathyroid suppression and postpartum neonatal complications of hypocalcemia, specifically seizures and tetany.^1^ Symptoms of hypercalcemia in pregnancy are non-specific, including nausea, vomiting, polyuria, polydipsia, abdominal pain, and neurocognitive symptoms, many of which are difficult to distinguish from physiologic changes of pregnancy.

Hypercalcemia in pregnancy can result from either PTH-dependent mechanisms, including primary hyperparathyroidism and familial hypocalciuric hypercalcemia, or PTH-independent mechanisms, including PTHrP-mediated hypercalcemia of pregnancy, milk-alkali syndrome, abnormal vitamin D metabolism, malignancy, and drugs.^1^ Here we present a case of a rare cause of hypercalcemia in pregnancy, highlighting the challenges in diagnosis and management of this disease.

## Case presentation

A 41-year-old woman in her second ongoing pregnancy presented with anemia refractory to intravenous (IV) iron replacement. She was found to have hypercalcemia and renal impairment. Her past medical history was significant for nephrolithiasis requiring lithotripsy, upper extremity paresthesias with normal electromyography, depression, and anxiety. Her pregnancy history was significant for preterm prelabour rupture of membranes and preterm delivery at 34 + 2 weeks and 2 days of gestation with no reported gestational hypertension and a prior spontaneous miscarriage. Her medications included folic acid 1 mg daily and vitamin D supplementation 1000 IU daily, without any history of vitamin A supplementation or antacid use. There was no significant family history of nephrolithiasis or known genetic conditions; however, her sister had a history of hypercalcemia and mild renal impairment, with further work-up pending.

Her first trimester was complicated by hyperemesis gravidarum. The second trimester was notable for normocytic anemia with hemoglobin of 80 g/L and mean cell volume of 94 fL and acute kidney injury with creatinine of 137 μmol/L. She received three doses of intravenous (IV) iron sucrose for anemia with no increment in hemoglobin.

She was referred to Obstetric Internal Medicine for assessment at 22 weeks’ gestation for fatigue, restlessness, shortness of breath, and palpitations. She was found to have an elevated serum and ionized calcium of 3.08 mmol/L and 1.44 mmol/L, respectively, and a normal 24-h urine calcium of 5.0 mmol/day (reference 1.0–7.0 mmol/day), with undetectable PTH. Her hemoglobin remained low at 90 g/L, creatinine high at 99 mmol/L, phosphate 1.1 mmol/L, bicarbonate 22 mmol/L, ferritin elevated at 411 µmol/L, normal vitamin B12 levels, and negative hemolytic markers. Her prenatal supplements and vitamin D supplements were discontinued, and she was admitted to hospital for expedited work-up of her hypercalcemia.

A work-up for secondary causes of hypercalcemia was negative, including serum protein electrophoresis, urine protein electrophoresis (UPEP), serum free light chains, and angiotensin-converting enzyme levels. Thyroid function tests were normal with thyroid-stimulating hormone of 1.37 mU/L. MRI of the chest, abdomen, and pelvis was normal and medication review was non-contributory. Special approval for PTHrP assay was sought; however, it was repeatedly processed as PTH, and no PTHrP result was available. 1,25(OH)_2_D was elevated at 334 pmol/L (reference range 40–170 pmol/L), and 25(OH)D was normal at 192 nmol/L (reference range 75–200 nmol/L), though these results were not available during her pregnancy. She received normal saline for IV hydration and calcitonin at 4 IU/kg subcutaneous every 12 h for two doses with improvement, but not normalization, of her calcium. She was discharged home on IV fluids three times per week and referred to outpatient Adult Metabolics.

**Figure 1.**
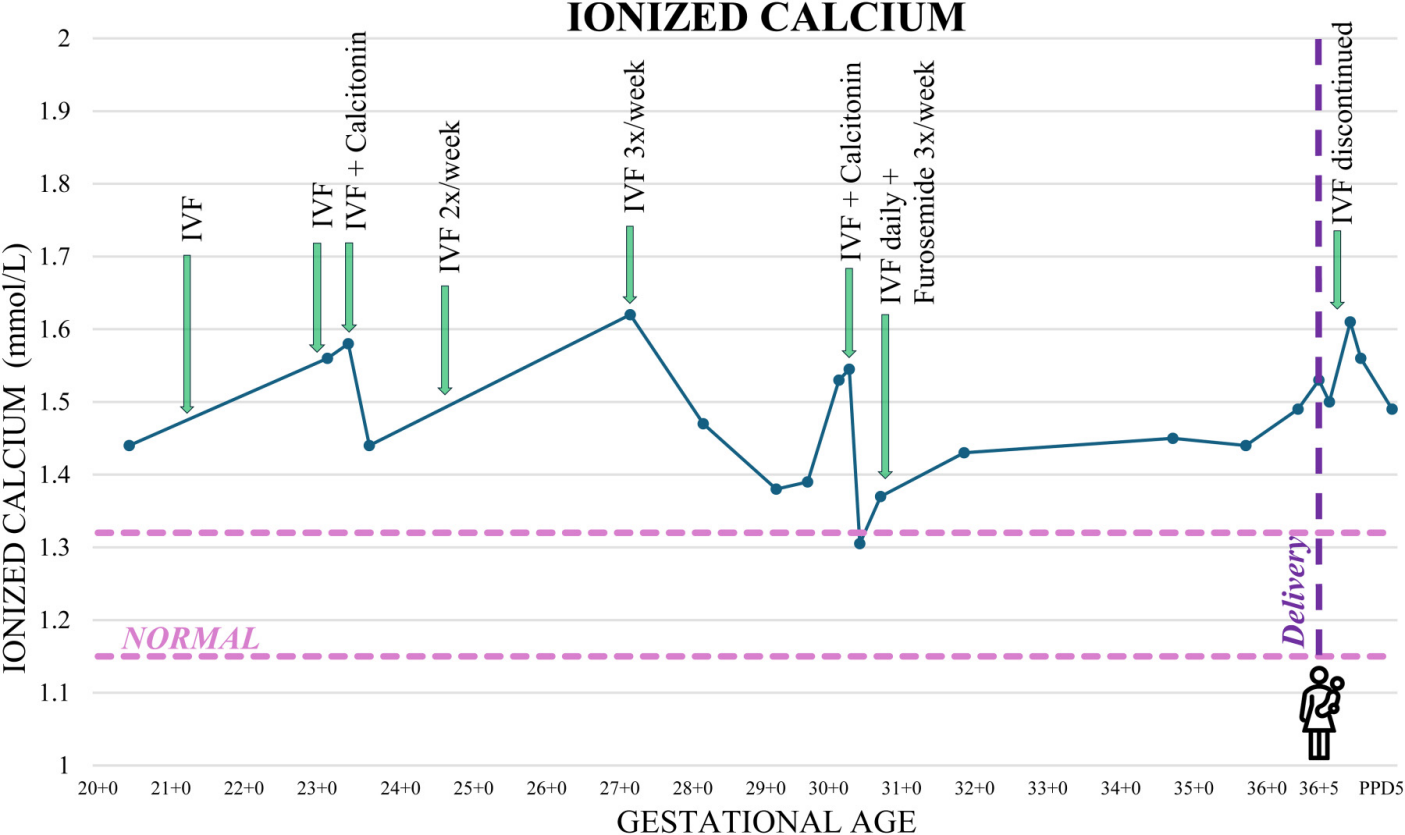
Maternal ionized calcium levels and timing of treatment interventions during pregnancy. IVF: intravenous fluids.

Despite lifestyle modifications, IV fluids, and calcitonin, she remained hypercalcemic (Figure 1). She required two additional doses of calcitonin again at 4 IU/kg, and IV normal saline was increased to daily with furosemide 10 mg IV three times weekly. She did not develop gestational hypertension; however, she did present with threatened preterm labor at 35 weeks with a spontaneous vaginal delivery at 36 + 5. The newborn had weekly calcium levels checked up to 4 weeks postpartum, all of which remained within normal limits, and had an uneventful postnatal course. Genetic testing was not performed in the newborn.

Postpartum, IV fluids were discontinued, and her serum calcium levels gradually normalized without intervention by 7 weeks postpartum. Her anemia had also resolved by 7 weeks postpartum, and renal function returned to baseline creatinine in the 90s within 1 week after delivery. Genetic testing of the *CYP24A1* gene revealed that she was homozygous for the pathogenic variant p.Glu143del. Following this result, her sister was evaluated for hypercalcemia, and genetic testing confirmed the same pathogenic homozygous variant.

## Discussion

The *CYP24A1* gene encodes 24-hydroxylase, which is responsible for inactivating 1,25(OH)_2_D and 25(OH)D. Loss-of-function mutations in *CYP24A1* were first identified in infants with idiopathic infantile hypercalcemia and have since been recognized to cause a spectrum of symptoms across all age groups.^5,6,7^

Previous studies have demonstrated an increase in 1,25(OH)_2_D during pregnancy primarily through renal, and to a lesser extent placental, upregulation of 1α-hydroxylase, independent of PTH. In the setting of 24-hydroxylase deficiency, this increase in 1,25(OH)_2_D may be exaggerated during pregnancy, leading to hypercalcemia.^8^ Furthermore, women may remain asymptomatic and normocalcemic until exposed to prenatal vitamins and physiologic changes of pregnancy, including increased uteroplacental PTHrP and vitamin D activation.^4,9,10^

The risk of hypercalcemia may be even greater in multiple pregnancies, likely due to increased 1α-hydroxylase production from two placentas and increased bone resorption via receptor activator of nuclear factor-kappa B ligand (RANKL) activation by 1,25(OH)_2_D.^10^ Aggressive management of hypercalcemia has been shown to improve both maternal and neonatal outcomes, reducing risk of preeclampsia, symptomatic hypercalcemia, and neonatal nephrocalcinosis.^8^ In our case, proactive treatment in the second pregnancy resulted in a later delivery compared to the index pregnancy.

Definitive diagnosis of *CYP24A1* mutations requires genetic testing, which can be challenging to access in a timely manner during pregnancy. A low 24,25(OH)_2_D and an elevated 25(OH)D: 24,25(OH)_2_D ratio strongly suggest reduced 24-hydroxylase activity, but these assays are rarely clinically available and thus unlikely to improve the diagnostic work-up.^11,12,13^

Management of hypercalcemia should begin with non-pharmacologic interventions, including hydration, avoidance of sunlight, dietary calcium reduction, and cessation of vitamin D and calcium supplements. Owing to the observational nature of available evidence, limited clinical data, and heterogeneous recommendations, there is no standardized approach to managing hypercalcemia in pregnancy secondary to pathogenic *CYP24A1* mutations, and pharmacologic options must take fetal exposure into account.

Loop diuretics, while recognized for their calciuric effect, also carry the risk of dehydration and potential worsening of hypercalcemia; therefore, their role in pregnancy remains uncertain. Calcitonin is considered safe during pregnancy at doses of 4–8 IU/kg, although its use may be limited by tachyphylaxis, which can develop after 48–72 h of continuous therapy.^1^ In our case, calcitonin and loop diuretics were used alongside IV fluids and were associated with a reduction, though not complete normalization, of calcium levels.

Bisphosphonates and denosumab, though effective postpartum, have traditionally been avoided in pregnancy due to teratogenicity and fetotoxicity concerns. A recent systematic review, in which the majority of women were exposed to bisphosphonates pre-conception and in the first trimester, found a congenital malformation rate of 3.8%, slightly above the population average of 3%. The types of congenital malformations were diverse and did not demonstrate a clear association between bisphosphonate use and a consistent anomaly pattern. Furthermore, the authors did not find increased rates of fetal loss, preterm birth, low birthweight, or neonatal hypocalcemia.^10,14^

Other targeted pharmacologic options include azole agents,which can reduce calcitriol synthesis; however, they are not routinely used inpregnancy due to potential teratogenicity and limited safety data.^15^ Rifampin, which induces 24-hydroxylase activity to increase catabolism of vitamin D metabolites, has a pregnancy safety profile supported by its use in managing latent tuberculosis.^16,17^ Glucocorticoids have been used, as they modulate vitamin D metabolism through the induction of 24-hydroxylation to deactivate vitamin D metabolites, as well as by reducing intestinal calcium absorption.

As summarized in Supplemental Table 3 of Cappelani et al., percentage reductions in serum calcium with various pharmacologic therapies are highly variable, and no single agent has demonstrated consistent efficacy.^18^ Given the complexity and potential for significant maternal and fetal morbidity, multidisciplinary management involving Maternal-Fetal Medicine, Obstetric Internal Medicine, and Adult Metabolics is essential.

This case highlights *CYP24A1* mutations as a rare cause of gestational hypercalcemia, unmasked by pregnancy physiology. With appropriate management, maternal and fetal outcomes are favorable.
